# Multienvironment Testing for Trait Stability and G × E Interaction on N_2_ Fixation, Plant Development, and Water-Use Efficiency of 21 Elite Groundnut (*Arachis hypogaea* L.) Genotypes in the Guinea Savanna

**DOI:** 10.3389/fpls.2019.01070

**Published:** 2019-09-12

**Authors:** Richard Oteng-Frimpong, Felix D. Dakora

**Affiliations:** ^1^Department of Crop Sciences, Tshwane University of Technology, Pretoria, South Africa; ^2^Department of Crop Improvement, CSIR-Savanna Agricultural Research Institute, Tamale, Ghana; ^3^Chemistry Department, Tshwane University of Technology, Pretoria, South Africa

**Keywords:** groundnut, stability, N_2_ fixation, mega-environment, additive main effects and multiplicative interaction, multienvironment trials, water-use efficiency

## Abstract

Groundnut production constitutes an integral part of the livelihoods of the people in the Guinea savanna of West Africa. This region accounts for over 70% of the total groundnut production in Ghana, 90% in Nigeria, and 100% in Mali and Burkina Faso. However, harsh environmental conditions often result in drastic yield reductions. In this study, we identified groundnut genotypes with superior symbiotic efficiency, greater pod yield, and plant water-use efficiency from 21 advanced groundnut breeding lines from ICRISAT after testing them at three locations in the Guinea savanna of Ghana over two consecutive years. Average N contribution by the groundnut genotypes ranged from 48 to 108 kg N ha^−1^, and mean pod yield from 580 to 2,100 kg ha^−1^. Genotype 17 (ICGV-IS 08837) produced about 2.5-fold more pods than genotype 1 (Chinese), which was the most widely cultivated variety by farmers. Of the 21 genotypes studied, genotype 16 (ICGV 99247) recorded the highest shoot δ^13^C value and was superior in water-use efficiency, which was consistent with stability estimates and mean performance. We also measured the effects of G × E on pod yield, N_2_ fixation, shoot δ^13^C, and mega-environments for testing groundnut in the Guinea savanna, and these were all significant, although the effect was minimal on shoot δ^13^C values. Of the locations studied, Nyankpala and Damongo were more discriminating, and each constituted a mega-environment for conducting future groundnut trials in the Guinea savanna. Genotype 3 (ICG 6222) emerged as the best cultivar for the Damongo mega-environment, while genotype 17 was the best genotype for the Nyankpala mega-environment. The genotypes exhibiting the highest sensitivity of N_2_ fixation in the environment included genotype 3 (ICG 6222), genotype 4 (ICGV 00068), and genotype 10 (ICGV 03315) (*b_i_* > 1.3), while *P_i_* estimates ranked genotypes 3, 10, and 17 as the best groundnut cultivars in terms of symbiotic N contribution. Based on the results of this study, genotype 17 (ICGV-IS 08837), genotype 3 (ICG 6222), genotype 10 (ICGV 03315), and genotype 4 (ICGV 00068), which were the most outstanding in terms of the overall pod yield, shoot biomass production, and amount of N-fixed, were the most suitable candidates to recommend for use in developing new varieties for the Guinea savanna of Ghana. Genotype 17 (ICGV-IS 08837) has already been released as a commercial variety for the Guinea savanna of Ghana since October 2018.

## Introduction

Groundnut (*Arachis hypogaea* L.) is an important food security crop cultivated throughout Ghana, with the Guinea savanna accounting for over 70% of total production (MoFA-SRID, 2014). About 90% of farming households in Ghana are involved in groundnut production (Tsigbey et al., 2003). In Africa, groundnut is the most important oilseed crop, usually grown for its edible oil (44 to 56%) and grain protein (22 to 30%), while the haulms also serve as a high protein fodder for livestock (Blümmel et al., 2012), especially in the Sahelian zones of Africa. In Ghana, groundnut is a major source of dietary protein for rural households, with sale of the grain also being a source of cash income (Martey et al., 2015).

Nodulated groundnut is reported to derive about 33–67% of its N nutrition from symbiosis and can contribute between 58 and 171 kg N ha^−1^ in South Africa (Mokgehle et al., 2014) and up to 101 kg N ha^−1^ in Northern Ghana (Dakora et al., 1987). The crop, therefore, has the potential to increase the N economy of soils in cropping systems.

However, grain yield on farmers’ fields is very low in the Guinea savanna, ranging from 0.8 to 1.2 t ha^−1^, which is less than the 3 t ha^−1^ obtained in China and the USA (FAO, 2014; MoFA-SRID, 2014). This yield gap has been attributed mainly to biotic stresses such as early and late leaf spot diseases caused by *Cercosporaarachidicola* S. Hori and *Cercosporidiumpersonatum* Berk. and Curt., respectively, as groundnut rosette disease (Nutsugah et al., 2007; Naab et al., 2009). Low soil fertility and intermittent drought together constitute the major abiotic stress (Hamidou et al., 2012; Vadez et al., 2012; Rademacher-Schulz et al., 2014).

Furthermore, climate change is predicted to result in reduced availability of arable land due to increased water scarcity and rise in temperature (Araus et al., 2014). Therefore, crop production per unit land area must be doubled to meet the food requirements of an ever-increasing population (Vadez et al., 2012; Araus et al., 2014). However, more importantly, crop genotypes that combine high tolerance to abiotic stress with yield stability are deemed to be more desirable by farmers.

Intermittent and terminal drought is experienced on farmers’ field due to the erratic distribution of rainfall in the Guinea savanna of Ghana. Therefore, genotypes that show tolerance to low moisture levels are more desirable for this region. Tolerance to low moisture availability has been studied using yield (pod/biomass) performance under stressed conditions. However, these traits are highly influenced by the environmental conditions and give inconsistent results (Nageswara Rao et al., 2001). The carbon isotope ratio (δ^13^C), which is a physiological trait, is less influenced by the environment and has been found to be a suitable surrogate for assessing water-use efficiency in groundnut (Wright and Nageswara Rao, 1993; Nageswara Rao and Wright, 1994; Wright et al., 1994). During photosynthesis, plants discriminate against ^13^C due to its heavier weight and slow diffusion to leaf surface. In effect, ^12^C is preferentially taken up by plants, resulting in less ^13^C in plants than in the atmospheric CO_2_ (O’Leary, 1988; Farquhar et al., 1989). The isotopic fractionation associated with the discrimination against ^13^CO_2_ (δ^13^C) during this process is a reliable indicator of the long-term water-use efficiency in C_3_ plants (Farquhar and Richards, 1984; Makoi et al., 2010; Mohale et al., 2014). The δ^13^C has been used to select groundnut genotypes for water-use efficiency under both field and glasshouse conditions (Nageswara Rao and Wright, 1994; Wright et al., 1994; Nageswara Rao et al., 1995; Sheshshayee et al., 2006; Rowland et al., 2012).

Cultivar development based on the assessment of the phenotypic values of different genotypes under varying environmental conditions is needed to select high-yielding and stable varieties (Marfo and Padi, 1999). However, genotypes tested in different locations or years often produce significant varying results in performance due to differences in response to factors such as soil fertility and/or presence of pathogens (Padi, 2007; Padi, 2008). This differential performance referred to as genotype × environment (G × E) interaction can complicate a genotype’s ability to demonstrate superior performance across environments (Fox et al., 1997; Romagosa et al., 2009). This is often manifested by a change in the performance ranking of genotypes or in the magnitude of differences between genotypes across environments (Romagosa et al., 2009). A significant G × E interaction is usually an indication that the effect of a genotype and an environment on a phenotype is not additive, thus resulting in inconsistent performance of genotypes across test sites (Anniccchiarico et al., 2005; Padi, 2008; Malosetti et al., 2013). Thus, the G × E interaction can result in low correlation between phenotypic and genotypic values, leading to a bias in heritability estimates and a slowdown in selection progress (Romagosa et al., 2009). In essence, the G × E interaction usually determines the optimum breeding strategy to adopt (Anniccchiarico et al., 2005; Romagosa et al., 2009).

Several methods can be used to examine G × E interaction (Becker and Leon, 1988; Fox et al., 1997; Malosetti et al., 2013), and these include components of variance analysis, stability analysis, as well as qualitative and multivariate analysis (Becker and Leon, 1988). However, the additive main effects and multiplicative interaction (AMMI) method is one of the most widely used (Romagosa et al., 2009; Malosetti et al., 2013), as it combines the analysis of variance of genotypes and the environment main effects with principal component analysis of the G × E interaction into one model (Gauch et al., 2008; Romagosa et al., 2009; Malosetti et al., 2013). The results of AMMI analysis are thus easily visualized in an interpretable and informative biplot that shows both main effects and G × E interaction (Malosetti et al., 2013). The genotype main effects plus G × E interaction (GGE) model is also effective in measuring and explaining G × E interaction (Gauch et al., 2008; Romagosa et al., 2009; Malosetti et al., 2013). As a tool, the GGE model with biplot display of the first two principal components is more popular among plant breeders (Yan, 2001; Malosetti et al., 2013), as it permits visualization of the data from different perspectives. The effect of G × E interaction on the performance of genotypes can be minimized by grouping fairly homogeneous locations into mega-environments in order to take advantage of specific adaptations (Gauch and Zobel, 1997). There is, however, no consensus on the use of the two models (AMMI and GGE), as they both have known limitations that are inherent to fixed effects models, including difficulty in treating variance heterogeneity and missing data.

The stability of agronomic traits and their contribution to plant growth and grain yield are important for cultivar recommendation (Becker and Leon, 1988), given that genotypes with high genetic potential are usually associated with low yield stability (Padi, 2004), a point that makes the goal of achieving high and stable yields elusive. The stability of genotypes can be measured using the coefficient of variability (*CV_i_*), genotypic variance (*S_i_^2^*), linear regression coefficient (*b_i_*), mean square deviation from the regression (*S^2^_di_*), stability variance (σ_i_^2^), ecovalence statistic (*W_i_*), and cultivar superiority estimate (*P_i_*) (Yates and Cochran, 1938; Finlay and Wilkinson, 1963; Eberhart and Russell, 1966; Shukla, 1972; Francis and Kannenberg, 1978; Lin et al., 1986; Lin and Binns, 1988). The *b_i_* estimate is considered as a type of dynamic stability where stable genotypes have no deviation from the general response to the environment. Genotypes with a *b_i_* value <0.7 are considered unresponsive to the environment, between 0.7 and 1.3 have average stability, and >1.3 are considered to have good response to the environment (Lin et al., 1986; Sudaric et al., 2006). The *P_i_* estimate is defined as the distance mean square between the cultivar’s response and the maximum response averaged over all locations, and a small mean square generally indicates superiority of the test cultivar (Lin and Binns, 1988; Lin and Binns, 1991).

There is a need to identify high-yielding new crop varieties adapted to the nutrient-poor soils and harsh climatic conditions of the Guinea savanna of Ghana. Unfortunately, however, effective cultivar selection is compounded by environmental variation and G × E interaction, which slow down the process. The aim of this study was to select superior groundnut genotypes with high and stable yields (though sometimes elusive), to identify mega-environments for groundnut selection in the Guinea savanna of Ghana, and find genotypes that are winners in the test environments.

## Materials and Methods

### Experimental Sites and Plant Materials

The experiments were conducted at Nyankpala, Yendi, and Damongo in the Guinea savanna of Ghana during the 2012 and 2013 cropping seasons. The Guinea savanna is characterized by a unimodal annual rainfall of 900–1,100 mm, which starts in May and ends in September/October each year (MoFA-SRID, 2014). The soil chemical characteristics, GPS coordinates, temperatures, and annual rainfall at the experimental sites in 2012 and 2013 are shown in **Table 1**.

**Table 1:** Environmental and soil characteristics of sites used in this study.

Environment	GPS coordinates	Total rainfall during trial	Temperature (˚C)		Total N	Organic C	Available P	Ca	S	pH
		(mm)	Min	Max	(%)	(%)	mg.kg^−1^	mg.kg^−1^	mg.kg^−1^	
Nyankpala 2012	9.3913, −1.0025	519.8	22.9	30.9	0.03	0.42	0.7	290	1.18	4.92
Yendi 2012	9.4978, −1.0239	518.6	22.8	31.0	0.05	0.60	4.3	551	1.45	6.23
Damongo 2012	9.0447, −1.8144	373.5	22.8	31.9	0.03	0.37	8.7	278	2.30	5.37
Nyankpala 2013	9.3913, −1.0025	608.3	23.2	31.4	0.02	0.32	8.0	232	2.40	4.40
Yendi 2013	9.4959, −1.0222	539.8	23.7	31.3	0.03	0.50	7.0	432	2.00	5.50
Damongo 2013	9.0439, −1.8156	504.8	22.8	30.9	0.02	0.37	12.0	232	2.10	4.70

Twenty-one groundnut genotypes comprising 17 advanced breeding lines from ICRISAT, 3 varieties from Ghana, and 1 variety from Nigeria (**Table 2**) were used in this study. Their pedigree, sources, and biological traits (maturity levels, leaf color, and seed coat color) are shown in **Table 2**. A randomized complete block design with four replicate plots was used. Each plot measured 3 m × 2 m with six rows. Inter- and intrarow spacings were 40 and 15 cm, respectively. The genotypes were sown in late July to early August in 2012 and 2013. No inputs (chemical fertilizers, rhizobial inoculants, etc.) were applied in this study. Weeds were controlled manually with a hoe, when necessary.

**Table 2:** Genotypes used in this study and their sources.

Code	Genotype	Pedigree	Subspecies	Botanical variety	Market class	Maturity class	Seed coat color	Leaf color score^#^	Source
1	CHINESE	Unknown	Fastigiata	Spanish	Runner	Early	Light tan	2	SARI, Ghana
2	ICG (FDRS) 4	ICGV 87157 (Argentine × PI 259747)	Hypogaea	Virginia	Virginia	Late	Tan	3	ICRISAT, Mali
3	ICG 6222	Germplasm line	Hypogaea	Virginia	Virginia	Late	Purple	5	ICRISAT, Mali
4	ICGV 00068	(ICGV 92069 × ICGV 94088) F2-SSD-SSD-B2-B1-B1(VB)	Hypogaea	Virginia	Virginia	Late	Purple	3	ICRISAT, Mali
5	ICGV 00362	(ICGV 86300 × ICGV 92242)	Fastigiata	Spanish	Runner	Medium	Pale tan	3	ICRISAT, Mali
6	ICGV 03166	(ICGV 87378 × ICGV 96342)	Fastigiata	Spanish	Runner	Early	Pale tan	2	ICRISAT, Mali
7	ICGV 03179	(ICGV 96300 × ICGV 96352)	Fastigiata	Spanish	Runner	Early	Tan	2	ICRISAT, Mali
8	ICGV 03196	(ICGV 96342 × ICGV 98266)	Fastigiata	Spanish	Runner	Early	Tan	2	ICRISAT, Mali
9	ICGV 03206	(ICGV 98191 × ICGV 93382)	Fastigiata	Spanish	Runner	Early	Light tan	3	ICRISAT, Mali
10	ICGV 03315	(ICGV 91284 × ICGV 87846)	Hypogaea	Virginia	Virginia	Early	Light tan	3	ICRISAT, Mali
11	ICGV 91317	(U4-7-5 × JL 24)	Fastigiata	Spanish	Runner	Early	Pale Tan	3	ICRISAT, Mali
12	ICGV 91324	(U4-7-5 × PI 337394F)	Fastigiata	Spanish	Runner	Early	Tan	2	ICRISAT, Mali
13	ICGV 91328	(J 11 × U4-7-5)	Fastigiata	Spanish	Runner	Early	Pale tan	3	ICRISAT, Mali
14	ICGV 97188	(ICGV 86887 × ICGV 87121)	Fastigiata	Spanish	Runner	Medium	Light tan	3	ICRISAT, Mali
15	ICGV 99029	(ICGV 94118 × ICGV 93427)	Fastigiata	Spanish	Runner	Late	Purple tan	4	ICRISAT, Mali
16	ICGV 99247	(ICGV 87354 × SANGDI)	Fastigiata	Spanish	Runner	Medium	Tan	3	ICRISAT, Mali
17	ICGV-IS 08837	(Argentine × PI 129747) F3	Fastigiata	Spanish	Runner	Medium	Tan	3	ICRISAT, Mali
18	ICIAR 19 BT	ICGM/754 × ICGV 87922	Fastigiata	Spanish	Runner	Early	Light tan	3	ICRISAT, Mali
19	KPANIELLI	Unknown	Fastigiata	Spanish	Runner	Late	Red	5	ICRISAT, Mali
20	NKATIESARI	F-mix × ICG (FDRS) 20	Fastigiata	Spanish	Runner	Medium	Dark tan	5	SARI, Ghana
21	SUMNUT 22	Unknown	Fastigiata	Spanish	Runner	Medium	Dark tan	3	Nigeria

^#^Scoring done using the leaf color chart (Witt et al., 2005).

### Data Collection

Pod yield was determined for plants harvested from a 1-m^2^ area at physiological maturity for all sites, except Yendi in 2012. The pods per unit area were air dried to a constant weight and pod yield (kg ha^−1^) estimated for each plot using plant density.

Plant biomass yield was assessed from weighing groundnut shoots dried at 60°C for 72 h. The dried shoots were milled and sieved through a 0.5-mm mesh. Subsamples of the ground powder were analyzed using mass spectrometry for ^15^N/^14^N and ^13^C/^12^C composition. The ^15^N/^14^N values were used to calculate the δ^15^N of each genotype using the following formula:

δ15N(‰)=[N15/N14]sample−[N15/N14]air[N15/N14]air×1000

Nonleguminous species (reference plants) growing on the plots at the time of biomass sampling were also collected and processed in a similar manner as the groundnut shoots. The reference plants used included *Celosia laxa*, *Euphorbia heterophylla*, *Hyptis suorelense*, *Zea mays*, *Panicum* spp., *Sorghum bicolor*, *Tridax procumbens*, *Cassia obstusifolia*, and *Andropogon gayanus*. The δ^15^N of these nonlegumes were also determined as described earlier. The mean δ^15^N value of the reference plants and individual groundnut shoots were used to calculate the percent nitrogen derived from the atmosphere (%Ndfa) and, finally, the amount of N-fixed by the different genotypes using the formula below (Shearer and Kohl, 1986; Unkovich et al., 2008);

$$\%\text{Ndfa}=\left(\frac{\delta^{15}{\text{N}}_{\text{ref}}-\delta^{15}{\text{N}}_{\text{leg}}}{\delta^{15}{\text{N}}_{\text{ref}}-{\text{B}}_{\text{value}}}\right)\times 100$$

where δ*^15^N_ref_* is the mean δ^15^N of nonleguminous species used as reference plants, δ*^15^N_leg_* is the δ^15^N value of the respective groundnut shoots, and the *B_value_* is the δ^15^N value of a groundnut plant that depended solely on atmospheric N_2_ for its N nutrition. For this study, the *B_value_* used was −1.35‰ (Unkovich et al., 2008).

The amount of N-fixed per plant was calculated using the following method proposed by (Maskey et al., 2001; Pule-Meulenberg et al., 2010):

$${\text{N-fixed}}_{\left(g/plant\right)}=\left(\frac{\%\text{Ndfa}}{100}\right)\times {\text{shoot biomass}}_{\left(\text{g/plant}\right)}$$

Shoot δ^13^C was calculated from the ^13^C/^12^C in each shoot sample using the formula below (Mohale et al., 2014);

δ13C (‰)=[C13/C12]sample−[C13/C12]standard[C13/C12]standard*1000

### Data Analysis

Analysis of variance (ANOVA) was done for each data set using GenStat v.12.1 (Payne et al., 2009). Homogeneity of variances was tested for all traits in the three locations in 2012 and 2013 using the Bartlett’s test.

The data for the three locations and two seasons were combined to give a total of six environments and analyzed using a two-way ANOVA. Genotypic ($\sigma_{\text{g}}^{2}$ ) and phenotypic ($\sigma_{\text{p}}^{2}$ ) variances were computed using the expected mean squares from the analysis of variance table, as described by Ntawuruhunga and Dixon (2010). Broad sense heritability (*H*^2^) was estimated (Padi, 2008) as

$$\begin{array}{c} H^{2}=\frac{\sigma_{\text{g}}^{2}}{\sigma_{\text{p}}^{2}}\\ \sigma_{\text{p}}^{2}=\sigma_{\text{g}}^{2}+\left(\frac{\sigma_{\text{ge}}^{2}}{E}\right)+\left(\frac{\sigma_{\text{e}}^{2}}{ER}\right) \end{array}$$

where $\sigma_{\text{g}}^{2}$ = genotypic variance, $\sigma_{\text{p}}^{2}$ = phenotypic variance, $\sigma_{\text{ge}}^{2}$ = genotype × environment variance, $\sigma_{\text{e}}^{2}$ = pooled error, *E* = number of environments, and *R* = number of replicates.

Estimates of the phenotypic coefficient of variation (PCV) and genotypic coefficient of variation (GCV) were obtained as described by Singh and Chaudhary (1979):

GCV(%)=√σg2'X'*100PCV(%)=√σp2'X'*100

where ‘X’ = grand mean of trait

Single-site analysis using residual maximum likelihood (REML) method was also done with genotypes as random effects to obtain the best linear unbiased predictions (BLUPs) of the mean for estimating variance components related to the genotypes and design factors using Breeding View v. 3.0 software (Breeding Management System v.3.0.1). The broad sense heritability for each trait was calculated from the variance components. The analysis was repeated with genotypes as fixed effects to obtain the best linear unbiased estimates (BLUEs) of the mean for use in the G × E interaction analysis. The G × E interaction was assessed with the AMMI2 model, and the scores of the first two principal components (IPCA 1 and IPCA 2) were plotted to visualize the G × E interaction on genotypes, as well as the adaptation of genotypes to the test environments. GGE biplot statistical software was used to visualize the mean versus stability and which-won-where biplot (Yan, 2001; Yan and Tinker, 2006). The correlation between environments was computed and the stability of each genotype over the environments calculated using the *b_i_*, and *P_i_* model.

## Results

### Analysis of Variance

Single site ANOVA (data not shown) revealed highly significant differences (p<0.05) among the genotypes for pod yield in each of the test environments (**Table 3**). The combined ANOVA revealed highly significant effects of genotypes, locations, years, and the interactions for all measured traits (**Table 4**). For all measured traits, locational effects accounted for the highest proportion of the total variation in the phenotype. In pod yield, location accounted for 55% of the total sum of squares, with genotype and G × E interaction accounting for only 14 and 15% of the total sum of squares, respectively, for pod yield (**Table 4**). Location effect on shoot biomass was high (34% of total sum of squares), while genotypic effect was moderate (14% of total sum of squares). The interactive effect of G × E on shoot biomass was moderate and accounted for only 16% of the total sum of squares (**Table 4**). The amount of N-fixed was highly influenced by location, which accounted for 47% of the total sum of squares. However, genotype and G × E interaction on N-fixed was moderate and accounted for 12.7 and 12.8%, respectively, of the total sum of squares (**Table 4**). Shoot δ^13^C values were largely influenced by the genotype (36% of total sum of squares) and the location (29% of total sum of squares), while only 9% of the variation was attributable to G × E interaction (**Table 4**).

**Table 3:** Mean pod yield (kg ha^−1^) of groundnut genotypes evaluated in the guinea savanna of Ghana between 2012 and 2013.

Serial no.	Genotype	Location				
		Damongo 2012	Nyankpala 2012	Nyankpala 2013	Yendi 2013	Damongo 2013
1	CHINESE	1,172.0 eh	627.5 bf	1,116.7 b	471.8 f	699.0 h
2	ICG (FDRS) 4	1,651.0 be	952.5 b	798.1 c	811.2 c	2,643.0 a
3	ICG 6222	2,849.0 a	847.0 bd	124.7 fh	680.8 de	2,424.0 ab
4	ICGV 00068	*nd*	645.0 bf	224.0 fh	463.8 f	2,502.0 ab
5	ICGV 00362	*nd*	793.5 be	202.6 fh	432.1 fh	891.0 h
6	ICGV 03166	1,112.0 fh	530.5 cf	44.2 gh	155.8 kl	2,444.0 ab
7	ICGV 03179	927.0 gh	590.0 bf	644.9 cd	300.3 hj	1,524.0 eg
8	ICGV 03196	1,280.0 dh	641.0 bf	282.4 eg	211.5 jl	1,828.0 ce
9	ICGV 03206	874.0 h	468.5 df	469.2 de	155.1 kl	1,296.0 g
10	ICGV 03315	1,990.0 b	511.0 cf	281.1 eg	440.7 fg	2,013.0 cd
11	ICGV 91317	1,106.0 fh	417.5 ef	42.3 gh	370.2 fi	2,614.0 a
12	ICGV 91324	1,413.0 cg	420.5 ef	56.1 gh	102.9 l	1,770.0 de
13	ICGV 91328	1,375.0 cg	579.0 bf	340.4 ef	317 gj	1,475.0 eg
14	ICGV 97188	1,821.0 bc	679.0 bf	154.8 fh	246.8 ik	1,370.0 fg
15	ICGV 99029	*nd*	897.0 bc	102.6 fh	302.6 hj	1,843.0 ce
16	ICGV 99247	1,271.0 dh	353.0 f	*nd*	79.8 l	1,329.0 fg
17	ICGV-IS 08837	*nd*	1,553.0 a	2,670.2 a	1,358 a	2,790.0 a
18	ICIAR 19BT	827.0 h	757.0 be	1,185.3 b	401.6 fh	1,377.0 fg
19	KPANIELLI	1,440.0 cf	555.5 cf	134.9 fh	782.1 cd	1,698.0 df
20	NKATIESARI	1,723.0 bd	399.0 ef	1,111.2 b	1,180.1 b	1,940.0 cd
21	SUMNUT 22	798.0h	452.0 df	242.9 eh	648.4 e	2,160.0 bc
	s.e	301.2	233.6	148.8	83.95	232.3
	CV%	26.8	35.9	30.6	17.8	12.6

Means followed by different letters are significantly different at p < 0.05. nd, not determined; CV, coefficient of variation; s.e, standard error.

**Table 4:** ANOVA of pod yield, shoot biomass, N-fixed, and shoot δ^13^C values of 21 groundnut genotypes tested at three locations over 2 years.

Source of variation	d.f.	Pod yield		Biomass		N-fixed		δ^13^C	
		m.s.	% Var	m.s.	% Var	m.s.	% Var	m.s.	% Var
Genotype (G)	20	1.75***	13.8	13.14***	14.0	3,762.40***	12.7	2.25***	35.5
Location (L)	2	70.16***	55.4	313.46***	33.5	122,867.00***	47.2	18.42***	29.0
Year (Y)	1	1.99***	0.8	86.94***	4.7	12,473.60***	4.4	9.21***	7.2
L × Y	2 (1)	8.04***	3.2	2.51**	0.3	3,316.50***	0.6	2.35***	3.7
G × L	40	0.92***	14.5	7.31***	15.6	1,844.00***	12.8	0.27***	8.5
G × Y	20	1.09***	8.6	9.13***	9.8	2,730.00***	9.2	0.38***	6.1
G × L × Y	40 (15)	0.63***	3.8	6.51***	13.9	1,990.60***	13.1	0.32***	10.0
Residual	375 (297)	0.04779		0.4087		103		0.1314	

***p < 0.001, **p < 0.01, d.f., degree of freedom (values in brackets apply to only pod yield); m.s., mean squares; % var, percentage of total sum of square.

### Analysis of G × E Effect Using AMMI Model

The AMMI model partitioned the total G × E effect into two principal components (IPCA1 and IPCA2, **Table 5**). For pod yield, IPCA1 and IPCA2 were both highly significant (*p <* 0.001) and accounted for 82% of the total G × E interaction. With shoot biomass, both IPCA1 and IPCA2 were again significant (*p <* 0.001 and *p* < 0.01, respectively) and accounted for 79% of the total G × E interaction. About 87% of the total G × E interaction affecting N-fixed was explained by IPCA1 and IPCA2 values, which were both highly significant (*p* < 0.001). For shoot δ^13^C, IPCA1 and IPCA2 were both significant (*p* < 0.01) and together accounted for 62% of the total G × E interaction.

**Table 5:** ANOVA table for AMMI model applied to pod yield, shoot biomass, N-fixed, and shoot δ^13^C values of groundnut genotypes tested in six environments.

Source of variation	d.f.	Pod yield (kg ha^−1^)		Biomass (kg ha^−1^)		N-fixed (kg ha^−1^)		δ^13^C (‰)	
		m.s.	% Var	m.s.	% Var	m.s.	% Var	m.s.	% Var
Genotypes	20	2.927		13,140,093		3,762		2.255	
Environments	5 (4)	32.679		143,779,384		52,968		10.15	
Interactions	100 (75)	0.635		7,353,814		2,080		0.313	
IPCA 1	24	1.113***	56.07	17,577,393***	57.36	4,052***	46.75	0.479***	36.78
IPCA 2	22	0.568***	26.24	7,327,203***	21.92	3,764***	39.81	0.355***	25.01
Residuals	54 (29)	0.291		2,820,843		518		0.221	

***p < 0.001, d.f., degree of freedom (values in brackets apply to only pod yield); m.s., mean squares; % var, percentage of total interaction sum of squares.

### Genetic Variation in Traits and Variance Components

**Figure 1:**
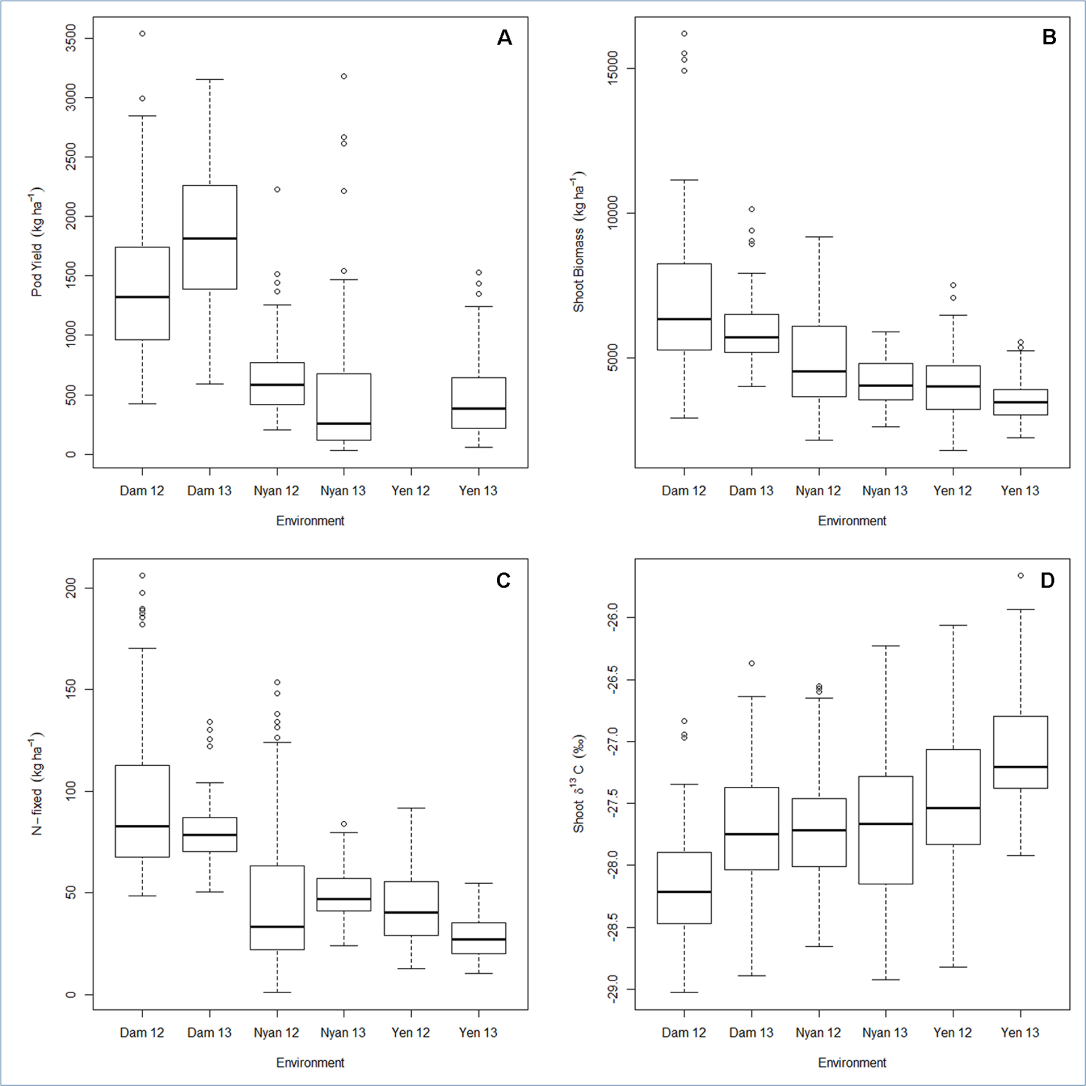
Box plot for **(A)** pod yield, **(B)** shoot biomass, **(C)** N-fixed, and **(D)** shoot δ^13^C displaying the total range, interquartile range, and median. Environment names are coded as Dam, Damongo; Nyan, Nyankpala; Yen, Yendi. The 12 refers to 2012, while 13 refers to 2013.

Pod yield ranged from 580 to 2,100 kg ha^−1^, with a mean of 960 kg ha^−1^ (**Table 6**). The genetic variation in pod yield was higher at Damongo in 2013 and lowest at Nyankpala in 2012 (**Figure 1A**). Shoot biomass ranged from 3,900 to 6,900 kg ha^−1^, with a mean of 4,950 kg ha^−1^ (**Table 6**). The genetic variation in shoot biomass was higher at Damongo in 2012 and lowest at Yendi in 2013 (**Figure 1B**). The amount of N-fixed ranged from 48 to 108 kg ha^−1^, with a mean of 64 kg ha^−1^ (**Table 6**). The extent of variation in N-fixed was highest at Nyankpala in 2012 and lowest at Nyankpala in 2013 (**Figure 1C**). Shoot δ^13^C values also ranged from −28.0 to −26.8‰, with a mean of −27.62‰ (**Table 6**). The genetic variation in shoot δ^13^C was highest at Nyankpala in 2013 and lowest at Damongo in 2013 (**Figure 1D**).

**Table 6:** Estimates of mean, range, variance components, broad sense heritability, and genotypic and phenotypic coefficient of variation for selected traits of 21 groundnut genotypes grown in six environments.

Trait	Mean	Range	σ^2^_g_	σ^2^_ge_	σ^2^_e_	σ^2^_p_	H^2^	GCV	PCV
Pod yield (kg ha^−1^)	960	580–2,100	731	2,156	4,094	1,367	0.53	2.81	3.85
Biomass (kg ha^−1^)	4,950	3,900–6,860	0.84	1.74	0.41	1.29	0.65	18.48	22.90
N-fixed (kg ha^−1^)	64.0	48–108	0.009	0.018	0.004	0.012	0.73	26.94	31.45
Shoot δ^13^C (‰)	−27.62	−28.0–−26.8	0.102	0.045	0.131	0.114	0.89	1.15	1.22
Shoot δ^15^N (‰)	1.87	0.07–3.86	0.098	0.211	0.054	0.136	0.72	16.72	19.66

σ^ 2^_g_, genotypic variance; σ^ 2^_ge_, genotype × environment variance; σ^ 2^_e_, error variance; σ^ 2^_p_, phenotypic variance; H^2^, broad sense heritability; PCV, phenotypic coefficient of variation; GCV, genotypic coefficient of variations.

The G × E interaction variance (σ^2^_ge_) accounted for the highest phenotypic variance (σ^2^_p_) for the traits studied (**Table 6**). With the exception of pod yield, σ^2^_ge_ was higher than genotypic variance (σ^2^_g_) and error variance (σ^2^_e_) for shoot biomass, amount of N-fixed, and shoot δ^13^C. Broad sense heritability estimates were 65% for shoot biomass, 72% for shoot δ^13^C 73% for amount of N-fixed, and a moderate 53% for pod yield. Genotypic coefficient of variation (GCV) ranged from 1.15% for shoot δ^13^C to 26.94% for amount of N-fixed. However, phenotypic coefficient of variation was moderate for shoot δ^13^C and pod yield (>10%) but high for shoot biomass and N-fixed (>20%).

### Mean Versus Stability and Stability Analysis

The average environments coordinate (AEC) of the GGE biplot compared the means of genotypes relative to their stability. AEC–GGE biplots were generated for pod yield, shoot biomass, amount of N-fixed, and shoot δ^13^C values in groundnut grown at six environments (**Figure 1A–D**, **Supplementary Figure 1**).

The mean pod yield of the genotypes in each environment are presented in **Table 3**. Genotype 17 (ICGV-IS 08837) was the best in pod yield on the AEC–GGE biplot, with genotype 21 (SUMNUT 22) being just above average (**Supplementary Figure 1A**). However, genotype 20 (NKATIESARI) and genotype 2 [ICG (FDRS) 4] successfully combined high pod yield with stability, with genotype 17, genotype 2, ICGV 6222, genotype 10 (ICGV 03315), and genotype 20 also displaying potential for superior pod yield when compared to the rest (**Supplementary Figure 1A**). Of the latter group, genotype 17 exhibited the least sensitivity to the environment (*b_i_* = 0.61), followed by genotypes 20 and 2, which were moderately sensitive (*b_i_* = 0.76 and 1.23, respectively). The remaining genotypes with high pod-yielding potential were very sensitive to the environment (*b_i_* > 1.3). Of all the genotypes tested, genotype 1 (CHINESE) showed the least sensitivity (*b_i_* = 0.07) to the environment for pod yield. Cultivar superiority estimates confirmed the high-yielding genotypes as the best materials for increased groundnut production in the Guinea savanna of Ghana.

Shoot biomass accumulation was highest in genotypes 3 and 4 (ICGV 00068) (6,860 and 6,270 kg ha^−1^, respectively), a point confirmed by the AEC-GGE biplot, even though genotype 3 showed higher stability than genotype 4 (**Supplementary Figure 1B**). Only three genotypes (genotypes 3, 4, and 17) showed high sensitivity to the environment (*b_i_* > 1.3) with regards to accumulation of shoot biomass (**Supplementary Table 1**); other genotypes were less or moderately sensitive to environmental factors. The *P_i_* estimates identified genotypes 3 and 4 as exhibiting the best plant growth and greater shoot biomass accumulation.

Amount of N-fixed was highest for genotypes 3 and 17 (108 and 88 kg N ha^−1^, respectively), a superiority confirmed by the AEC–GGE plot, which showed that only 6 out of the 21 genotypes evaluated recorded above average symbiotic performance (**Supplementary Figure 1C**). The genotypes exhibiting the highest sensitivity of N_2_ fixation to the environment included genotypes 3, 4, and 10 (*b_i_* > 1.3), with the remaining 18 genotypes showing low to moderate sensitivity of the symbiosis to the environment. In this study, *P_i_* estimates ranked genotypes 3, 10, and 17 as the best groundnut cultivars in symbiotic N contribution (**Supplementary Table 1**).

With shoot δ^13^C, genotypes 2, 5 (ICGV 00362), and 14 (ICGV 97188) all had *b_i_* values >1.3, with only genotype 3 showing <0.7 *b_i_* value. The remaining genotypes showed moderate sensitivity (*b_i_ <*1.3 to >0.7) of shoot δ^13^C to the environment. However, the *P_i_* estimates ranked genotypes 16 (ICGV 99247) (−26.80‰), genotype 5 (−27.15‰), and genotype 2 (−27.28‰) with greater δ^13^C values or higher water-use efficiency than the remaining genotypes (**Supplementary Table 1**).

Of all the traits studied, mean performance ranking was positively correlated to *P_i_* ranking, but negatively correlated to *b_i_*. Pod yield was the only trait with *P_i_* and *b_i_* rankings that were not significantly correlated.

### Identification of Mega-Environments and Best Expressed Traits in Genotypes

Based on pod yield, two mega-environments were identified for groundnut in the Guinea savanna of Ghana (**Supplementary Figure 2A**). One mega-environment comprised two environments which were Damongo 2012 and Damongo 2013. Of the 21 genotypes tested, genotype 3 emerged as the best cultivar for this mega-environment. The second mega-environment identified in terms of pod production consisted of Nyankpala 2012, Nyankpala 2013, and Yendi 2013, and here, genotype 17 emerged as the best groundnut genotype. In contrast, three mega-environments were identified based on shoot biomass production (**Supplementary Figure 2B**). Nyankpala 2012 and Nyankpala 2013 environments were placed in two different mega-environments. Genotype ICIAR 19 BT was the best genotype in biomass accumulation in the Nyankpala 2013 mega-environment, while genotype 5 emerged as the best in biomass production in the Nyankpala 2012 mega-environment. The third mega-environment in terms of shoot biomass comprised Yendi 2012, Yendi 2013, Damongo 2012, and Damongo 2013, with genotype 4 producing the highest biomass.

Two mega-environments were identified in the Guinea savanna of Ghana for the amount of N-fixed by groundnut (**Supplementary Figure 2C**). The first mega-environment consisted of four environments, namely, Nyankpala 2012, Damongo 2012, Damongo 2013, and Yendi 2013, with genotype 3 as the highest in symbiotic N contribution. The second mega-environment comprised Yendi 2012 and Nyankpala in 2013, with genotype 4 as the best in symbiotic N yield.

With δ^13^C, all the six tests used in this study were grouped together to form one mega-environment (**Supplementary Figure 2D**). In this cluster, genotype 16 exhibited the highest δ^13^C value (or water-use efficiency), a finding consistent with stability estimates.

## Discussion

The effect of G × E interaction on any trait is observable when genotypes differentially perform across environments (Malosetti et al., 2013). The G × E interaction therefore poses a challenge to effective selection during breeding as it can reduce the heritability of traits in different environments (Romagosa et al., 2009). A genotype with a high mean performance and a low G × E interaction is usually considered suitable for a larger environment. However, when G × E interaction is high, genotypes may be selected for local adaptation (Gauch and Zobel, 1997). To ensure high yield stability and economic returns in the Guinea savanna, superior groundnut genotypes should be adapted to a broad range of environmental conditions. However, to avoid risk, farmers want genotypes that always win, as it is difficult to achieve consistent yields under their growing conditions (Padi, 2008). This means that information on G × E interaction and yield stability ought to be critical for any groundnut breeding program in the Guinea savanna.

In this study, the genotypic variance, environmental variance, and G × E interaction variance for the groundnut genotypes were highly significant for pod yield, shoot biomass, N-fixed, and shoot δ^13^C values. Although the genotype main effect for pod yield, shoot biomass, and N-fixed was also highly significant, it contributed <15% to the total sum of squares, in contrast to the genotype main effect for δ^13^C, which contributed 35% of the total sum of squares. This suggested that environmental influence on shoot δ^13^C values was low, justifying the need for the use of this physiological trait in the identification of drought-tolerant groundnut genotypes in multienvironment field trials (Nageswara Rao et al., 2001; Arunyanark et al., 2008). The large location effect on pod yield, biomass, and N-fixed reported in this study is consistent with the study of Gauch and Zobel (1996), who reported that a median yield trial has ∼70% of variation in E, 20% in GEI, and 10% in G, clearly indicating that the location effect is always larger than the other components.

While it may be argued that the plot size sampled for yield analysis in this study is small, optimum plot size is a function of soil/field heterogeneity. Breeding programs make use of single row plots to limit the space requirement for experiments and reduce heterogeneity in experimental plots (Upadhyaya et al., 2005; Oteng-Frimpong et al., 2019). Using small plot sizes to extrapolate yield on a per ha^−1^ basis is not new, especially when dealing with a large number of genotypes.

Marfo and Padi (1999) found strong contributions by genotype and location main effects as well as their interaction to variation in pod yield of 12 groundnut accessions evaluated in the Guinea savanna of Ghana. The G × E interaction was also found to significantly influence the yield of 47 groundnut genotypes in the same savanna environment (Padi, 2008). The findings of this study are therefore consistent with those of Marfo and Padi (1999) and Padi (2008) and thus underscored the strong effect of G × E interaction on groundnut production in the Guinea savanna. The highly significant year × location and year components of the total sum of squares emphasized that both the predictable environment (location) and the unpredictable environment (year) sources of variation were important in the performance of groundnut in the Guinea savanna (Adugna and Labuschagne, 2002). This, however, demonstrated the difficulty in selecting superior groundnut genotypes for such an environment.

A major contributor to genotypic variation in this study was the differences in soil fertility across the test environments and the spatial variability, as there was no plot-to-plot variation in plant density. As shown in **Table 1**, the soil sampled from Damongo in 2012 contained 300 mg N kg^−1^ and 8.7 mg kg^−1^ of available P, while the soil from Yendi contained 500 mg N kg^−1^ and 4.3 mg kg^−1^ of available P. High soil N can inhibit N_2_ fixation in groundnut (Mokgehle et al., 2014), while high levels of plant-available P enhances plant growth and N_2_ fixation in legumes, including groundnut (Bhadoria et al., 2002; Naab et al., 2009; Ohyama et al., 2011; Mohamed and Abdalla, 2013; Jat and Meena, 2014; Tanabata and Ohyama, 2014). It is therefore understandable that plant growth, N_2_ fixation, and pod yield were higher at Damongo (low soil N and higher P) than Yendi, where soil N was higher and P lower. An example was seen in genotype 12 (ICGV 91324), which fixed 74 kg ha^−1^ N at Damongo, while at Yendi, it fixed only 22 kg ha^−1^. Although it can be argued that plant growth and symbiotic performance with zero inputs (rhizobial inoculants, chemical fertilizers, etc.) at each site in this experiment was due to variation in native rhizobial strains and mineral nutrients across sites, the study directly mimicked farmer practice in the region.

Rainfall was another important contributor to the variation in plant growth, N_2_ fixation, and pod yield of groundnut genotypes across the test environments. Although the total amount of rainfall recorded during the trials seemed enough to support optimal plant growth (**Table 1**; Guled et al., 2013), the distribution was poor and had a negative effect on N_2_ fixation and pod yield (Kumar et al., 2012). For example, at Nyankpala, there was a break in rainfall for over 25 days in 2013 (just before flowering), a drought event that resulted in soil water deficit. The plants were thus exposed to temporary drought which impacted negatively on pod yield (100 kg ha^−1^) of genotype 6 (ICGV 03166), genotype 11 (ICGV 91317), and genotype 12. However, these same genotypes produced >1,700 kg ha^−1^ pod yield at Damongo, a location that experienced relatively better rainfall distribution during the same 2013 cropping season. The temporary drought imposed as a result of the poor rainfall distribution could also explain the high variability in shoot δ^13^C values observed at Nyankpala (Craufurd et al., 1999; Anyia and Herzog, 2004; Songsri et al., 2013).

Although the mean maximum daily temperature difference between environments was <1°C, its differential effect on crop plant growth, and hence contribution to G × E interaction, cannot be ignored (Prasad et al., 2000). In fact, small differences in vapor pressure deficit (not measured in this study) at study sites elsewhere contributed differentially to plant growth and yield, and this might have resulted in the observed G × E interaction (Kumar et al., 2012).

Using a limited number of seasons and their interactions to define mega-environments for identifying best performing genotypes can be fraught with risk if the planting season is not representative of the location, so an alternative to that is the use of crop stimulation models which take into consideration longer-term climate changes (Chenu, 2014). However, in this study, the planting seasons were quite representative of the locations. It is also important to note that genotype 1, which was the recommended variety used by farmers in the early 1980s, proved inferior to many genotypes in this study, especially genotype 17. The declined yield of genotype 1 cultivated since the 1980s indicated why breeding programs must continue to release new varieties to replace old ones that exhibit declining yields.

The observed high shoot δ^13^C values of genotype 16 at all the test environments clearly indicated its greater water-use efficiency and hence drought tolerance relative to the other genotypes (Wright et al., 1988; Wright et al., 1994; Condon et al., 2004). Increased water-use efficiency can be achieved either through greater photosynthetic capacity, a reduction in stomatal conductance, or both (Condon et al., 2002; Condon et al., 2004). However, a reduction in stomatal conductance usually results in decreased photosynthesis and consequently decreased biomass accumulation. This probably explained why, in this study, genotype 16, which exhibited greater water-use efficiency, had low shoot biomass and pod yield. The drought tolerance genes of genotype 16 could therefore be used to improve genotypes 17, 3, and 20, which recorded higher shoot biomass and greater pod yield but displayed lower water-use efficiency by adopting conventional backcrossing and/or marker-assisted backcrossing.

In this study, the AMMI2 model proved useful in partitioning the G × E interaction as the first two principal components could explain more than 80% of the total G × E interaction for all the traits studied. The Nyankpala and Damongo environments accounted for a greater proportion of the total G × E interaction for pod yield, shoot biomass, and amount of N-fixed. Interestingly, these two locations were also the most discriminatory for the same traits, as they fell relatively far away from the AEC axis (**Figures 1A–D**, **S3**). Soil fertility was better at Damongo compared to Nyankpala and Yendi, and this probably accounted for the observed differences in genotypic performance in the contrasting locations. in Addition, rainfall at Nyankpala was more erratic compared to Damongo and Yendi. Thus, the poor rainfall distribution at Nyankpala in 2013 could be responsible for the marked variation in shoot δ^13^C at that site compared to the other locations. The differences in environmental conditions at these two locations might have accounted for the observed high G × E interaction effect on the genotypes.

Heterogeneity of genotypic variances among and between environments, as well as a lack of genetic correlation among environment variances, have been observed in the presence of significant G × E interaction (Padi, 2008; Crossa, 2012; Malosetti et al., 2013). The significant G × E interaction in this study can be largely attributed to the low correlation between and among environments for all the tested traits except shoot δ^13^C (**Supplementary Figure 3**). Such low correlations do not permit the selection of genotypes for much wider adaptation as the environments are not only unrelated but also independent of each other (Gauch and Zobel, 1997). Creating relatively homogenous environments (mega-environments) within the region allows the identification of genotypes for adaptation to specific environments for higher superior performance (Gauch and Zobel, 1997; Gauch et al., 2008). In this study, two mega-environments were identified for pod yield and amount of N-fixed, with each year at Nyankpala separating out as a mega-environment and Damongo plus Yendi grouping together as one mega-environment for the two traits.

In this study, the stability of the 21 groundnut genotypes was assessed using the Finlay–Wilkinson regression slope (*b_i_*) and cultivar superiority estimates (*P_i_*). The *P_i_*estimates ranked genotypes 17, 3, 2, 20, and 10 as the most superior and stable cultivars for shoot biomass, pod yield, and amount of N-fixed. Genotype mean rankings and *P_i_*rankings of genotypes displayed a strong correlation between the two parameters. The high correlation when combined with the high heritability of *P_i_* estimates further confirmed that these genotypes were really superior for those traits (Lin and Binns, 1988; Lin and Binns, 1991) and are likely to be the most suited for environments with improved crop management and favorable climatic conditions (Makinde et al., 2013).

From the which-won-where biplots (**Supplementary Figures 2A–D**) genotype 3 seemed more adapted to Damongo, while genotype 20 was more adapted to Nyankpala and Yendi. Interestingly, genotype 1, which is the most widely cultivated groundnut variety in the Guinea savanna of Ghana ranked 14th for mean pod yield, but this was compensated for by its relatively good stability. In this study, genotypes 16, 5, and 2 showed low stability but exhibited much higher shoot δ^13^C values, a clear indication of their greater water-use efficiency and relative drought tolerance. However, genotype 16 seemed more adapted to Damongo and Yendi, while genotype 5 was more adapted to Nyankpala. The fact that some genotypes were stable for one trait but unstable for another suggested that the genetic factors involved in the G × E interaction differed between traits (Stagnari et al., 2013).

Taken together, the findings of this study seemed to suggest that different groundnut genotypes should be recommended for each study site. This argument is consistent with the recommendations of previous studies on groundnut bred for the Guinea savanna of Ghana. Furthermore, the AEC–GGE biplots showed that future selections for superior groundnut genotypes can be effectively done at Nyankpala and Damongo. A plot of the raw means across the environments against their variances (**Supplementary Figure 4**) showed that the GGE model used in the study was adequate. Genotype 16, which recorded greater shoot δ^13^C and hence higher water-use efficiency, would be the ideal candidate for use in future hybridization programs to improve the water-use efficiency and drought tolerance of groundnut in Ghana’s Guinea savanna. Furthermore, genotypes 17, 3, 10, and 4, which ranked highest for overall pod yield, shoot biomass production, and amount of N-fixed, could be recommended for further studies aimed at developing new varieties for the Guinea savanna of Ghana. Given the moderate to high heritability estimates of the traits assessed in this study, high genetic gains should be expected in a future hybridization program.

## Author Contributions

FD conceptualized the study and edited the manuscript. RO-F carried out the experiment and wrote the draft paper. FD supervised the doctoral study of RO-F, which was included in this work. All authors read and contributed to finalize the manuscript.

## Funding

This study was supported with funds from the Bill and Melinda Gates Foundation (BMGF) under the auspices of the BMGF-Project on Capacity Building in Africa (awarded to Tshwane University of Technology, Pretoria). RO-F is grateful to the Foundation for a competitive doctoral fellowship awarded under the Foundation with BMGF-Project, to CSIR-Savanna Agricultural Research Institute, Ghana, for grant of a study leave, and to ICRISAT for seed material and training support. The DST/NRF South African Research Chair in Agrochemurgy and Plant Symbioses and the Tshwane University of Technology are duly acknowledged for their continued funding support of FD’s research.

## Conflict of Interest Statement

The authors declare that this study was conducted in the absence of any commercial or financial relationships that could be construed as a potential conflict of interest.
