# Supplementary material for: Multienvironment Testing for Trait Stability and G × E Interaction on N2 Fixation, Plant Development, and Water-Use Efficiency of 21 Elite Groundnut (Arachis hypogaea L.) Genotypes in the Guinea Savanna
Source: Front Plant Sci. 2019 Sep 12;10:1070. doi: 10.3389/fpls.2019.01070 (PMC6751404; doi:10.3389/fpls.2019.01070)
Supplement: Supplementary file 1 [file DataSheet_1.docx]

Supplementary Table 1: Stability of pod yield in 21 groundnut genotypes tested at three locations over two years

|  |  |  | **Pod yield (t ha^-1^)** | | |  | **Biomass (t ha^-1^)** | | |  | **N-fixed (kg ha^-1^)** | | |  | **Shoot δ^13^C (‰)** | | |
| --- | --- | --- | --- | --- | --- | --- | --- | --- | --- | --- | --- | --- | --- | --- | --- | --- | --- |
| **Serial no.** | **Genotype** |  | **Mean** | ***b_i_*** | ***P_i_*** |  | **Mean** | ***b_i_*** | ***P_i_*** |  | **Mean** | ***b_i_*** | ***P_i_*** |  | **Mean** | ***b_i_*** | ***P_i_*** |
| 1 | CHINESE |  | 0.82 | 0.07 | 1.124 |  | 4.65 | 0.57 | 11.1 |  | 57 | 0.75 | 3044 |  | -27.66 | 0.81 | 0.47 |
| 2 | ICG (FDRS) 4 |  | 1.37 | 1.23 | 0.562 |  | 5.17 | 0.69 | 9.1 |  | 67 | 0.73 | 2649 |  | -27.28 | 1.36 | 0.19 |
| 3 | ICG 6222 |  | 1.39 | 1.76 | 0.757 |  | 6.86 | 1.74 | 2.9 |  | 108 | 2.15 | 110 |  | -28.00 | 0.53 | 0.90 |
| 4 | ICGV 00068 |  | 0.96 | 1.58 | 0.935 |  | 6.27 | 3.33 | 3.1 |  | 79 | 2.39 | 1811 |  | -27.34 | 1.18 | 0.20 |
| 5 | ICGV 00362 |  | 0.58 | 0.37 | 1.169 |  | 5.36 | 1.18 | 7.2 |  | 59 | 1.29 | 2555 |  | -27.15 | 1.39 | 0.14 |
| 6 | ICGV 03166 |  | 0.86 | 1.50 | 1.252 |  | 4.31 | 0.92 | 11.9 |  | 53 | 0.87 | 3261 |  | -27.73 | 0.90 | 0.52 |
| 7 | ICGV 03179 |  | 0.80 | 0.68 | 1.144 |  | 4.31 | 0.37 | 13.6 |  | 57 | 0.54 | 3290 |  | -27.75 | 0.93 | 0.55 |
| 8 | ICGV 03196 |  | 0.85 | 1.09 | 1.124 |  | 4.45 | 0.31 | 12.8 |  | 55 | 0.49 | 3314 |  | -27.75 | 0.91 | 0.56 |
| 9 | ICGV 03206 |  | 0.65 | 0.67 | 1.36 |  | 3.90 | 0.69 | 14.3 |  | 48 | 0.66 | 3784 |  | -27.96 | 0.87 | 0.87 |
| 10 | ICGV 03315 |  | 1.05 | 1.36 | 0.898 |  | 5.65 | 1.28 | 6.4 |  | 77 | 1.47 | 1254 |  | -27.45 | 0.94 | 0.30 |
| 11 | ICGV 91317 |  | 0.91 | 1.56 | 1.224 |  | 4.57 | 0.56 | 11.7 |  | 60 | 0.75 | 2584 |  | -27.35 | 0.94 | 0.27 |
| 12 | ICGV 91324 |  | 0.75 | 1.26 | 1.28 |  | 4.72 | 0.56 | 12.1 |  | 62 | 0.81 | 2592 |  | -27.74 | 1.09 | 0.59 |
| 13 | ICGV 91328 |  | 0.82 | 0.89 | 1.136 |  | 5.06 | 0.57 | 10.9 |  | 73 | 0.64 | 2169 |  | -27.63 | 1.17 | 0.49 |
| 14 | ICGV 97188 |  | 0.85 | 1.03 | 1.14 |  | 4.23 | 0.34 | 14.0 |  | 51 | 0.53 | 3624 |  | -27.49 | 1.30 | 0.36 |
| 15 | ICGV 99029 |  | 0.79 | 1.13 | 0.971 |  | 5.66 | 0.98 | 6.8 |  | 61 | 1.06 | 2757 |  | -27.96 | 1.05 | 0.79 |
| 16 | ICGV 99247 |  | 0.76 | 0.98 | 1.571 |  | 4.34 | 1.19 | 10.7 |  | 56 | 1.31 | 2682 |  | -26.80 | 0.99 | 0.01 |
| 17 | ICGV-IS 08837 |  | 2.03 | 0.61 | 0.115 |  | 5.50 | 1.49 | 6.2 |  | 80 | 1.26 | 1496 |  | -27.89 | 0.98 | 0.79 |
| 18 | ICIAR 19BT |  | 0.91 | 0.32 | 0.984 |  | 4.43 | 0.48 | 13.4 |  | 60 | 0.42 | 3423 |  | -27.90 | 0.80 | 0.75 |
| 19 | KPANIELLI |  | 0.92 | 0.97 | 1.093 |  | 5.05 | 1.51 | 7.6 |  | 63 | 0.99 | 2870 |  | -27.82 | 1.03 | 0.64 |
| 20 | NKATIESARI |  | 1.27 | 0.76 | 0.578 |  | 5.15 | 0.85 | 8.6 |  | 63 | 1.05 | 2741 |  | -27.77 | 0.80 | 0.58 |
| 21 | SUMNUT 22 |  | 0.86 | 1.06 | 1.221 |  | 4.35 | 0.50 | 12.8 |  | 56 | 0.60 | 3234 |  | -27.69 | 1.00 | 0.52 |


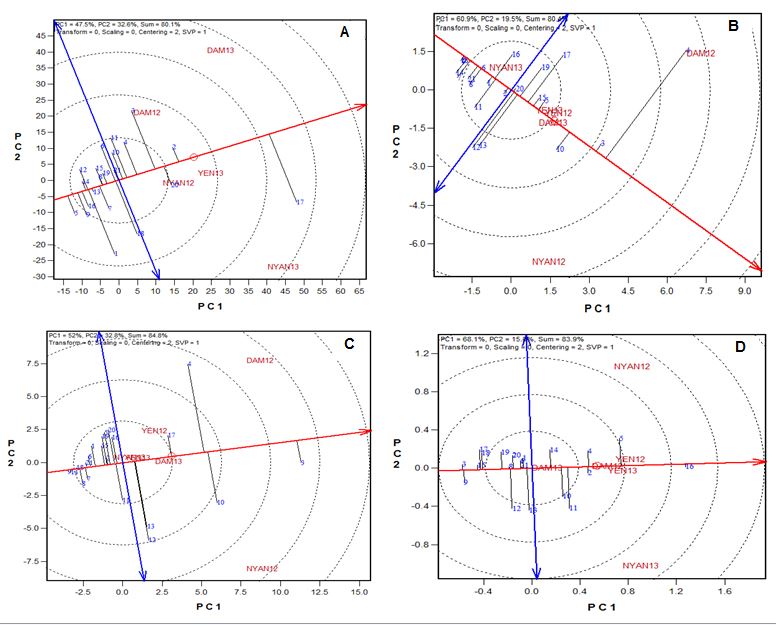


Supplementary Figure 1: Average-Environment Coordination (AEC) view showing the mean performance and stability of genotypes for A) pod yield (kg ha^-1^), B) shoot biomass (kg ha^-1^), C) N-fixed (kg ha^-1^) and D) shoot δ^13^C (‰). Genotype names are represented by numbers 1 – 21 as shown in Table 2. Environment names are coded as Dam = Damongo, Nyan = Nyankpala and Yen = Yendi. The 12 refers to 2012 while 13 refer to 2013.


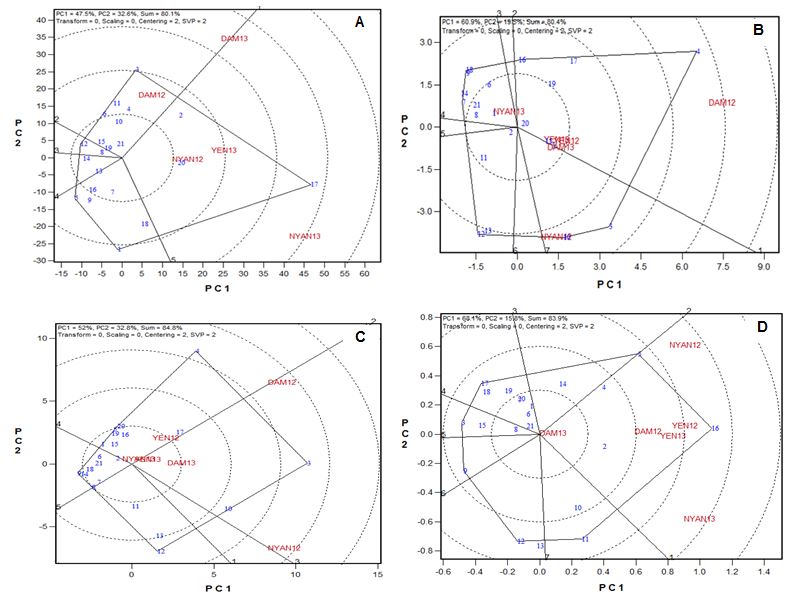


Supplementary Figure 2: Which-won-where biplot displaying the best genotypes and mega environments for A) pod yield (kg ha^-1^), B) shoot biomass (kg ha^-1^), C) N-fixed (kg ha^-1^) and D) shoot δ^13^C (‰). Genotype names are represented by serial numbers 1-21 as shown in Table 2. Environment names are coded as Dam = Damongo, Nyan = Nyankpala and Yen = Yendi. The 12 refers to 2012 while 13 refer to 2013.


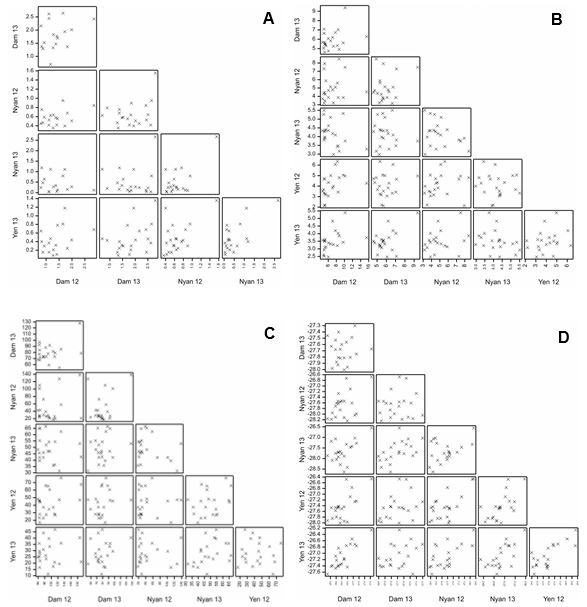


Supplementary Figure 3: A scatter plot matrix displaying the correlation between six environments for A) pod yield (kg ha^-1^), B) shoot biomass (kg ha^-1^), C) N-fixed (kg ha^-1^) and D) shoot δ^13^C (‰). Environment names are coded as Dam = Damongo, Nyan = Nyankpala and Yen = Yendi. The 12 refers to 2012 while 13 refer to 2013.


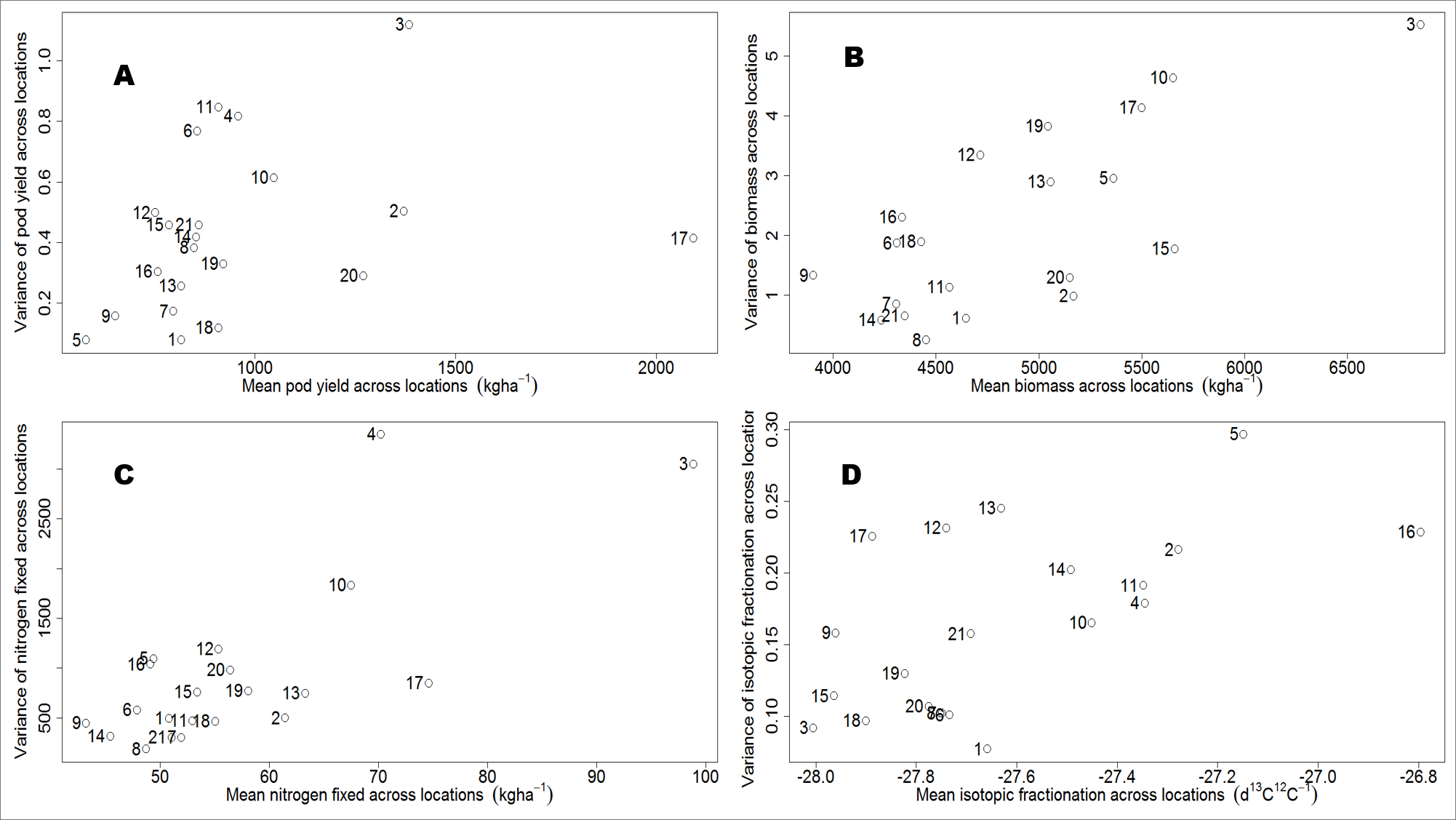


Supplementary Figure 4**:** Mean vs. variance plot across locations for A) pod yield, B) biomass, C) N-fixed and D) δ^13^C (Lin et al. 1986).
